# Benefits of Virgin Coconut Oil in Diet to *Colossoma macropomum* (Cuvier, 1818)

**DOI:** 10.1155/2022/4387692

**Published:** 2022-10-19

**Authors:** Márcia Valéria Silva do Couto, Natalino da Costa Sousa, Higo Andrade Abe, Joel Artur Rodrigues Dias, Carlos Alberto Martins Cordeiro, Peterson Emmanuel Guimarães Paixão, Thays Brito Reis Santos, Fernanda dos Santos Cunha, Juliana Oliveira Meneses, Ricardo Marques Nogueira Filho, Carol Nunes Costa Bomfim, Cláucia Aparecida Honorato, Bruno Trindade Cardoso, Rodrigo Yudi Fujimoto

**Affiliations:** ^1^Department of Animal Science, Post-Graduation Progam, Federal University of Pará, Castanhal, Brazil; ^2^Department of Health and Environment, Post-Graduation Program, Tiradentes University, Aracaju, Brazil; ^3^Department of Fishing Engineer, Laboratory of Nutrition, Federal University of Sergipe, Aracaju, Brazil; ^4^Department of Agricultural Science, Federal University of Grande Dourados, Mato Grosso do Sul, Brazil; ^5^Brazilian Agricultural Research Corporation, Embrapa Tabuleiros Costeiros, Aracaju, Sergipe, Brazil

## Abstract

This study investigated the effects of different dietary levels of virgin coconut oil on growth, body composition, bacterial resistance, and hematology parameters in tambaqui (*Colossoma macropomum*). Six isolipidic (12% crude lipid) and isonitrogenous (33% CP) diets were formulated adding virgin coconut oil (0%, 25%, 50%, 75%, and 100%) as lipid source, replacing the soybean oil. A positive control diet also prepared containing 15% lauric acid (main fatty acid in virgin coconut oil). Triplicate groups of 20 fish were fed twice daily throughout 90 days. Monthly, we evaluated the tambaqui growth performance, weight and biomass gain, specific growth ratio, apparent feeding conversion, relative condition factor, fish weight uniformity, and final survival. At end of experiment, the fish were subjected to bacterial challenge and blood analysis (glucose, lactate, plasmatic protein, and red cell blood). Fish fed 0%, 100% of VCO and lauric acid presented lower growth than fish fed 50% of virgin coconut oil (VCO) which presents the highest biomass (929.8 ± 80.6a) and weight gain (15.4 ± 4.3a) (*p* < 0.05). Furthermore, the fish fed 50% and 75% VCO had an increase on body protein (50 and 58%, respectively) without increase body fat content. The values of triglycerides and cholesterol decreased (242.4 ± 39.1c and 181.5 ± 14.6bc) in fish fed 50% VCO and lauric acid, respectively. After bacterial challenge, a hemolytic anemia occurred in fish submitted to diets containing 100% of soybean oil and 100% of VCO, causing 41.67% and 100% of mortality, respectively. However, fish fed with 25 and 50% of VCO not presented any clinical signs of disease or mortality. In conclusion, dietary inclusion of 50% virgin coconut in substitution to soybean oil as a lipid source in diets for *C. macropomum* is recommended to improve the growth performance, body protein, and resistance against pathogenic bacteria *Aeromonas hydrophila*.

## 1. Introduction

Aquaculture production depends on specific diets to provide a balanced nutrient profile, mainly in essential amino acids and fatty acids, to improve health and fish growth [1–3]. Many vegetable oils such as repassed oil, palm oil, and coconut oil are considered a promising source of lipids and energy, replacing fish oil, reducing costs, and improving fish performance (Hossein [4, 5]).

The virgin coconut oil (VCO) is an oil rich in medium-chain fatty acid, composed mainly by caprylic acid and lauric acid, which improve animal health and growth [6, 7]. In fish, the use of virgin coconut oil in diet to *Clarias gariepinus* (40%) improved growth performance and resistance against *Aeromonas hydrophila* ([8]). The benefits in immune response were related to the main metabolite of lauric acid, monolaurin, as well as monocaprilin and monocaprin [9].

Despite the benefits, scientific reports about benefits of replacement of soybean oil for virgin coconut oil for tropical fish are scarce. Among tropical fish, the tambaqui (*Colossoma macropomum*) was highlighted due its characteristics for captivity as good growth, acceptance of different diets, and high market value.

The specie is an endemic fish of Amazonian region, intensively reared in countries of South America [10], with a peculiar characteristic of its natural feeding habit which is constituted by fruits and seeds [11].

The tambaqui exhibits high lipid digestibility for seeds and fruits (>90%) and high requirement of lipid in juvenile phase (12% g/100 g, Rodrigues et al., 2018) and can naturally metabolize saturated fatty acids without affecting their body composition or health, making it a positive aspect to include VCO in the diet to tambaqui [12]. Thus, the study evaluated the inclusion of virgin coconut oil in the diet for tambaqui juveniles *Colossoma macropomum* on growth, body composition, blood parameters, and resistance against pathogenic bacterium *Aeromonas hydrophila*.

## 2. Material and Methods

### 2.1. Animals and Rearing System

Juveniles of *Colossoma macropomum* were acquired from Development Corporation of Vale do São Francisco (CODEVASF), Porto Real do Colégio, Alagoas, Brazil. The fish were distributed in polyethylene tanks (450 L) coupled to water recirculation system, at a stocking density of 1 kg/m^3^ for 10 days of acclimation [13]. All fish received industrial feed (Purina Nutripiscis®, 36% of crude protein) for seven days *ad libitum* twice a day.

Afterwards, the fish (3.03 ± 0.65 g; 5.64 ± 0.50 cm) were distributed to polyethylene tanks (150 L) at a stocking density of 20 fish per tank. The tanks were connected to mechanical and biological filter and supplied by artificial aeration. The fish were fed with experimental diets twice a day (09 and 17 h) at a feeding rate of 5% live weight (adapted from [14]) for 90 days. The animals remained at natural photoperiod and water quality maintained ideal for tambaqui rearing: temperature (27.1 ± 1.3°C), dissolved oxygen (7.2 ± 0.5 mg/L), pH (6.5 ± 0.3), and total ammonia (0.15 ± 0.10 mg/L).

### 2.2. Feeding Trial

#### 2.2.1. Experimental Diet and Fish Rearing

A control diet containing 33% crude protein and 12% lipid, using soybean oil as the major lipid source, was made following the nutrient requirements for tambaqui ([15–17] (Table 1). The experimental diets were composed of four levels of replacement of soybean oil with virgin coconut oil (25, 50, 75, and 100% VCO) and one diet containing lauric acid (15%, positive control, equivalent to a concentration of 25% VCO). The ingredients were mixed, pelletized (mod.PCP, 10 L. Poli), dried in an air-forced oven (60°C), and stored in a freezer (20°C).

Centesimal composition of the diets was carried out according to AOAC [18]: crude protein by Kjeldahl method, fat with Soxhlet using petroleum ether, and ashes and crude fiber according to Thiex [19]. The crude energy was calculated based on caloric values of lipid (9 kcal/g), protein (4 kcal/g), carbohydrate (4 kcal/g), ashes (2 kcal/g), and crude fiber (2 kcal/g) [20] (Table 1).

The fatty acid profile was determined using 100 mg of lipids dissolved in 2 mL of petroleum ether with 0.2 mL of a methanolic solution of KOH 2 mol/L vigorously mixed for 30 s in a tube shaker and then washed with a saturated NaCl solution [21]. The samples were analyzed using a gas chromatograph (Thermo Scientific Trace 1310) equipped with an automatic sampler (Thermo Scientific TriPlus) coupled to a mass spectrometer (Thermo Scientific Single Quadrupole ISQ QD). Fatty acids were separated in a TR-FAME column (70% cyanopropyl polysylphenylene-siloxane) with dimensions of 60 m × 0.25 mm ID × 0.25 *μ*m film thickness (Thermo Scientific, USA). The carrier gas was helium at a constant flow rate of 1.0 mL/min.

The temperature programming used in the chromatographic assay was as follows: the column oven temperature was initially held at 45°C, with a hold time of 2 min; then increased to 175°C at a rate of 10°C/min, remaining at that temperature for 20 min; and finally increased to 5°C/min up to 215°C, remaining at this temperature for 5 min. The conditions used in the mass spectrometer were a mass range of 40-440 *μ*m and ion source and interface temperatures of 250°C and 275°C, respectively.

Fatty acid methyl esters were identified in scan mode, and quantification was performed in SIM mode (selected ion monitoring) targeting the base ions at *m*/*z* = 74 (saturated acid esters), *m*/*z* = 55 (esters of monounsaturated acids), *m*/*z* = 67 (esters of di-unsaturated acids), and *m*/*z* = 79 (esters of polyunsaturated acids).

The components were identified based on the fatty acid profile of soybean, coconut, and corn oils [22] and mass spectra and retention times compared to fatty acid methyl esters present in the Supelco 37-component FAME mix standard (Supelco, USA) and in the NIST library available in the Xcalibur software. The peak area of each ester was normalized and expressed as a percentage relative to the sum of the areas of all peaks [23] (Table 2).

The experiment was conducted in a completely randomized design with six treatments in triplicates. At the end of the experiment, all fish were anesthetized and subjected to a biometric procedure to determine growth performance. The experiment was approved by the ethical committee of EMBRAPA, no. 0020.2018.

#### 2.2.2. Growth Performance, Biochemical Parameters, and Body Composition

Growth parameters such as biomass gain (BG (1)), weight gain (WG (2)), apparent feeding conversion (AFC (3)), specific growth ratio (SGR (4)), relative condition factor (Kr (5)), fish weight uniformity (*U* (6)) [24], and final survival (*S* (7)) were determined.

(1) $$\begin{array}{c} \text{BG}=\left(\text{fb}-\text{ib}\right), \end{array}$$

where fb is final biomass and ib initial biomass.

(2) $$\begin{array}{c} \text{WG}=\left(\text{fw}-\text{iw}\right), \end{array}$$

where fw is final weight and iw initial weight.

(3) $$\begin{array}{c} \text{AFC}=\left(\frac{\text{rc}}{\text{bg}}\right), \end{array}$$

where rc is ration consumption and bg biomass gain.

(4) $$\begin{array}{c} \text{SGR}=\frac{\;\ln\left(\text{fw}\right)-\ln\left(\text{iw}\right)}{\Delta t}\times 100, \end{array}$$

where fw is final weight, iw initial weight, and Δ*t* experimental time in days.

(5) $$\begin{array}{c} \text{Kr}=\left(\text{ow}-\text{ew}\right), \end{array}$$

where ow is observed weight and ew estimated weight.

(6) $$\begin{array}{c} U=\frac{N\pm 20\%}{\text{Nt}}\times 100, \end{array}$$

where *N* = total of fish with mean weight ± 20% and Nt is total of fish.

(7) $$\begin{array}{c} S=\left(\text{fs}-\text{is}\right)\times 100, \end{array}$$

where fs is the number of fish at the final of experiment and is number of fish at the beginning of experiment.

After the feeding trial (90 days), blood samples (500 *μ*L/fish) were collected from 21 fish per treatment using sterile syringes containing ethylenediamine tetraacetic acid (EDTA 3%). Aliquots of blood (10 *μ*L) were used to determine glucose (g/dL), triglycerides, and cholesterol (mg/dL) using quick test strips Accu-Chek Active® for glucose and quick test strips Accutrend Plus® for triglycerides and cholesterol. The total plasma protein (g/dL) was determined using the refractometer Quimis®.

After blood sampling, 2 fish per tank were euthanized by deepening of anesthesia (eugenol 60 mg/L) followed by medullar section, and then, the liver, spleen, and total viscera were removed to determine the hepatosomatic (liver weight/fish weight^∗^100), spleen somatic (spleen weight/fish without viscera), and viscerosomatic indices (viscera weight/fish weight^∗^100), respectively.

Afterwards, the fish was dried (55°C per 24 hours) and grounded (using grinder mod. PCP 10 L. Poli and Knife mill mod. TE 650.Tecnal) to determine body composition (crude protein, lipid, and ashes) according to AOAC (2016).

#### 2.2.3. Bacterial Challenge

The pathogenic bacterium *Aeromonas hydrophila* (CPQBA22808 DRM) was acquired from the laboratory of shrimp culture at the Federal University of Santa Catarina. The strain was grown in brain heart broth containing fish blood (1%) for 24 h at 30°C [25], centrifuged at 1800 × g, and the pellet was suspended in sterile saline solution at a concentration of 1 × 10^8^ CFU/mL [26, 27].

After the feeding trial, 4 fish from each tank (0, 25, 50, 75, and 100% of VCO) were reallocated into 10 L tanks at a stocking density of four fish per tank in quadruplicate. The animals received an intraperitoneal injection of *Aeromonas hydrophila*1 × 10^8^ CFU/g and were observed for ten days for clinical signs and mortality. Sixteen fish without VCO supplementation were also used to inject sterile saline solution, and another 16 fish were not injected. Clinical signs such as skin depigmentation, erratic swimming, petechial hemorrhages, lethargy, pale gills, fin erosion, and ulcerations were evaluated [26–29] The intensity of infection was classified according to the adapted methodology of Fishbein et al. [30], following grades 0 to 5.

Throughout the trial challenge, water quality parameters such as temperature (26.00 ± 01.00°C), pH (7.00 ± 0.20), dissolved oxygen (4.25 ± 0.70 mg/L), and ammonia (0.70 ± 0.15 mg/L) were monitored using multiparameter YSI.

#### 2.2.4. Blood Parameters after Bacterial Challenge

At the end of bacterial challenge (240 h), blood was collected of all survival fish to determine erythrocytes (cell/*μ*L) in the Neubauer chamber, hematocrit by the microhematocrit method, hemoglobin concentration (g/dL) (Kit Hemoglobin Bioclin), lactate (quick test strips Accutrend Plus®), glucose (quick test strips Accu-Chek Active®), total plasma protein (refractometer Quimis®), and hematological indexes: mean corpuscular volume (MCV (8)), mean corpuscular hemoglobin (MCH (9)), and mean corpuscular hemoglobin concentration (MCHC (10)).

(8) $$\begin{array}{c} \text{MCV}=\frac{\text{Ht}}{\text{Ery}}\times 10, \end{array}$$

(9) $$\begin{array}{c} \text{MCH}=\frac{\text{Hg}}{\text{Ery}}\times 10, \end{array}$$

(10) $$\begin{array}{c} \text{MCHC}=\frac{\text{Ht}}{\text{Hg}}\times 100. \end{array}$$

### 2.3. Statistical Analysis

All data were subjected to normality and homoscedasticity tests using the Shapiro–Wilk and Bartlett tests, respectively. Data without normality distribution were transformed into arcsine square roots (*x*/100) for survival and log (*x* + 1) for bacterial counting. Afterward, the data were analyzed by ANOVA followed by Tukey's test for mean comparison [31] using the statistical software Past 4.03.

## 3. Results

### 3.1. Feeding Trial

#### 3.1.1. Growth Performance and Body Composition

The inclusion of 50% VCO in the diet for tambaqui after 90 days of feeding promoted an increase in weight gain (15.4 ± 4.3a) and biomass gain (929.8 ± 80.6a). The lowest values of growth performance occurred for the treatments containing 100% VCO and lauric acid (positive control), followed by the negative control (100% soybean oil diet) (Table 3). No mortality occurred during the feeding trial.

**Figure 1:**
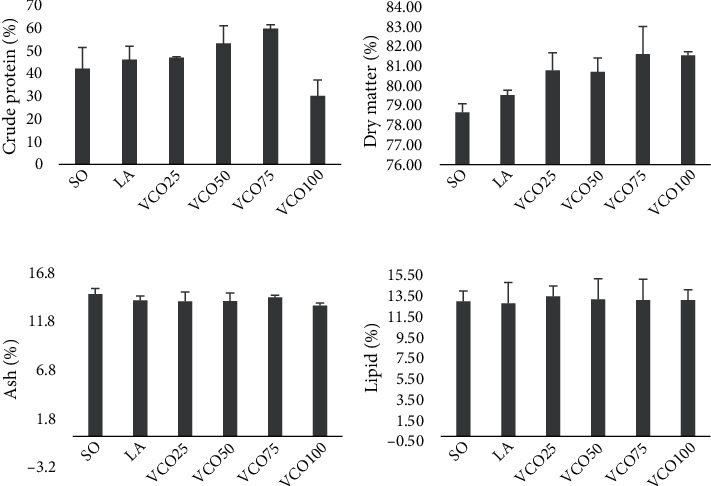
Body chemical composition of *Colossoma macropomum* submitted to different levels of virgin coconut oil and lauric acid: (a) crude protein, (b) dry matter, (c) ashes, and (d) lipids. Different letters in the columns mean statistical difference by the Tukey test (*p* < 0.05). SO: 100% soybean oil; LA: lauric acid; VCO: virgin coconut oil.

Regardless of the effects of VCO inclusion on growth performance, no statistical differences in somatic indices (Table 4) and body lipid content (Figure 1(c)) were observed among the treatments. Nonetheless, tambaqui fed diets containing 50% and 75% VCO showed the highest body protein values, contrary to the negative control and 100% VCO (Figure 1(a)).

In the feeding trial, there was an increase in glucose because of the inclusion of VCO in diets when compared to the soybean oil and lauric acid treatments. Furthermore, a reduction in cholesterol and triglyceride levels was observed in the lauric acid diet. However, higher levels of coconut oil (75 and 100%) increased these parameters, and contrary was observed in lauric acid and 25% and 50% levels of coconut oil. Total plasma protein did not show any statistical difference among the diets tested (Table 5).

#### 3.1.2. Bacterial Challenge

**Figure 2:**
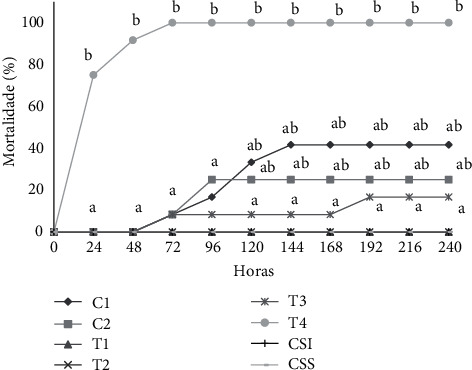
Accumulated mortality of the tambaqui *Colossoma macropomum* previously submitted to the dietary supplementation (90 days) containing different levels of virgin coconut oil (VCO: 25, 50, and 100%), lauric acid (LA), and soybean oil (SO) and fish without injection (FWI) and fish with saline solution (FWS) after bacterial challenge against *Aeromonas hydrophila* (fish were in observation for 240 hours to evaluate the mortality).

After the bacterial challenge, the group treated with 100% soybean oil (SO) had a mortality of 41.67%, which was higher than that of treatment with 25 and 50% of VCO, which did not present any clinical signs or mortality. The 75% VCO concentration resulted in a mortality of 16.67% after 192 h. Nonetheless, at a high concentration of 100% VCO, total mortality of the fish occurred (Figure 2).

**Figure 3:**
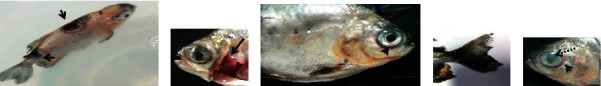
Clinical signs of tambaqui *Colossoma macropomum* after bacterial challenge with *Aeromonas hydrophila*: (a) dark spot and ulcerations (arrow), (b) pale gill (arrow), (c) hemorrhagic petechiae on skin (asterisk) and ocular region (arrow), (d) erosion fins, and (e) ocular opacity.

The most severe clinical signs, such as hemorrhagic petechiae (Figures 3(a) and 3(b)), pale gills (Figure 3(c)), darkest skin, fin erosion (Figure 3(d)), and ocular opacity (Figure 3(e)), were observed in fish fed 100% soybean oil, lauric acid, and 100% VCO (Table 6). The highest severity degree occurred in the treatment with 100% VCO, classified as the maximum level (degree 5%–94%) (Table 6).

The treatment containing 100% VCO concentration resulted in the highest glucose and total plasma protein values after infection with *Aeromonas hydrophila*. The treatments with 25% and 50% VCO presented the lowest values of lactate (Table 7).

The infection caused a reduction in erythrocyte values at concentrations of 25% and 100% VCO, lauric acid, and soybean oil treatments. The highest concentration of VCO resulted in the highest mean corpuscular volume but a lower mean corpuscular hemoglobin concentration (Table 8).

## 4. Discussion

The use of vegetables oils as lipid source in the fish diet can represent a profitable strategy for fish farmers directly influencing growth performance [32–34]. The inclusion of virgin coconut oil in the tambaqui diet improved the growth performance at inclusion of 50% VCO.

The tambaqui presents high trophic plasticity, reflected in specific mechanisms to reach its fatty acid requirement using desaturase and elongase enzymes to biosynthesize ARA, EPA, and DHA from C18 precursors in the diet, allowing this fish species to use different saturated and unsaturated oils as energy sources [15, 35–38].

Furthermore, VCO is a natural stimulant of digestive enzyme stearoyl-CoA desaturase that is extremely efficient in metabolizing saturated fatty acids [7, 39]. These facts explain the improvement in growth observed in this study, corroborating previous reports to *Clarias gariepinus* fed with 50 and 100% of virgin coconut oil [40], grouper (*Epinephelus coioides*) fed with 35% VCO diet [41], and tilapia *Oreochromis niloticus* fed diets containing 33% and 66% of coconut oil [42].

Despite the improvement in growth, there was no increase in viscerosomatic, spleen somatic, and hepatosomatic indexes, indicating that there is no existing storage of lipid or any metabolic problem related to inclusion of VCO in tambaqui diet. Several studies have reported that medium-chain fatty acids do not accumulate in adipose tissues [43–47]. There was also a reduction in triglycerides and cholesterol in fish fed with 50% of VCO, showing a direct influence on lipid metabolism by inclusion of virgin coconut oil in the diet. This reduction in triglycerides and cholesterol is mainly due to unsaponifiable compounds that influence fatty acid synthesis and oxidation rate, which decrease the enzymatic activity in cholesterol biosynthesis, increasing fatty acid catabolism [48].

The consumption of saturated fat acids is frequently associated to increases in cholesterol levels. Nonetheless, medium-chain fatty acid does not participate in chylomicron formation, reducing cholesterol level with benefits for the animal [43, 48–51]. Furthermore, the medium-chain fatty acid is not incorporated into lipoproteins and is catabolized in mitochondria without the need for carnitine [50, 52]. This characteristic reflected in a readily available energy source and consequently sparing high levels of glucose in fish submitted to dietary supplementation containing virgin coconut oil.

This better use of lipid from VCO promotes the protein-sparing effects, increasing the body protein in tambaqui fed with 50 and 75% VCO. These fatty acids are used as an alternative energy source, reducing glycogenesis and protein catabolism, allowing muscle formation [48, 53]. This protein-spare effect is due to medium chain fatty acid that produces ketone bodies from acetate, providing an alternative energy source to glucose [48]. Similar results were observed for *Clarias gariepinus* fed diets containing 50 and 100% VCO [24, 40, 53].

Corroborating the results of the feeding trial, fish exposed to pathogen bacteria fed 25 and 50% of virgin coconut oil showed no clinical signs or mortality, indicating better resistance against this illness. Saturated fatty acids can stimulate defense cells, increasing lymphocyte and neutrophil counts, as well as their phagocytic capacity [54, 55]. The lauric acid which is the main fatty acid component of VCO specifically influences the action of toll-like receptors on macrophages, making it more efficient against bacterial infection [56, 57]. Furthermore, the monolaurin, an acid lauric metabolite, is also able to solubilize lipids and phospholipids from bacterial cell membranes causing disintegration, inhibiting pathogen maturation, and preventing its binding to host cells [57, 58]. This increase in bacterial resistance due to monolaurin has already been reported in Nile tilapia against *Streptococcus iniae* infection [6, 39, 57].

Then, the adequate balance of fatty acids (saturated, monounsaturated, and polyunsaturated) from VCO and soybean oil provided the organism's homeostasis.

However, the highest oil concentration (100% of coconut oil) showed the worst growth performance in tambaqui probably due to the inadequate ratio of saturated : unsaturated fatty acid. This result corroborates the previous reports, where fish fed 100% coconut oil in the diet proved to be unsuitable for the growth of *Oncorhynchus mykiss* and *Labeo rohita* [59, 60]. In addition, after bacterial challenge, higher values of glucose and lactate and reduced values of red blood cells related to the stress caused by *Aeromonas hydrophila* infection were observed in the treatment with 100%VCO. These changes reflected in the classic clinical signs of aeromoniosis such as bleeding points and darkening skin (Figure 3) [61, 62] and the consequently high mortality observed in 100%VCO diet.

Reductions in the quantity and capacity of defense cells are commonly caused by dyslipidemia and/or excessive lipids in the diet, since excess of n-3 fatty acid family can depress n-6 metabolism, and vice versa, and the excess of n-6 can cause immunosuppression [42, 63–66]. Therefore, the dyslipidemia observed in 100%VCO treatment helped to reduce the response against the pathogen.

Thus, the use of virgin coconut oil proved to be a promising feed to promote protein-sparing effect by better lipid utilization for tambaqui rearing, when combined with another lipid source for adequate fatty acid balance.

## 5. Conclusion

The replacement of 50% soybean oil by virgin coconut oil as a lipid source in diets for *C. macropomum* is recommended to increase the productive performance, body protein, and resistance against *Aeromonas hydrophila*.

## Figures and Tables

**Table 1:** Ingredients and proximate composition of experimental diets containing different levels of virgin coconut oil and lauric acid.

**Ingredients (%)**	**Levels of VCO (%)**					
	SO (100%)	LA (15%)	VCO (25%)	VCO (50%)	VCO (75%)	VCO (100%)
Soybean meal	20.00	20.00	20.00	20.00	20.00	20.00
Corn meal	38.47	38.47	38.47	38.47	38.47	38.47
Fish meal	32.00	32.00	32.00	32.00	32.00	32.00
Soybean oil (SO)	8.00	6.00	6.00	4.00	2.00	0.00
Virgin coconut oil (VCO)	0.00	0.00	2.00	4.00	6.00	8.00
Vitamin C^1^	0.50	0.50	0.50	0.50	0.50	0.50
CMC^2^	0.00	0.84	0.00	0.00	0.00	0.00
Lauric acid	0.00	1.16	0.00	0.00	0.00	0.00
Vitamin and mineral mix	1.00	1.00	1.00	1.00	1.00	1.00
BHT^3^	0.03	0.03	0.03	0.03	0.03	0.03
Total	100.00	100.00	100.00	100.00	100.00	100.00
**Chemical composition**						
	SO (100%)	LA (15%)	VCO (25%)	VCO (50%)	VCO (75%)	VCO (100%)
Dry matter (%)	87.06	88.05	87.83	87.8	87.93	86.34
Crude protein (%DM)	32.81	33.12	33.39	33.49	32.71	33.45
Ashes (%DM)	8.12	7.64	8.12	8.16	7.88	8.16
Crude fiber (%DM)	3.19	3.29	3.34	3.43	3.21	3.32
Lipid (%DM)	12.1	11.1	12.8	12.6	12.5	12.0
Crude energy (kcal·kg^−1^ diet)	4422	4424	4444	4427	4434	4427

SO: soybean oil; LA: lauric acid; VCO: virgin coconut oil; BHT: butylated hydroxytoluene; DM: dry matter basis.

**Table 2:** Fatty acid profile present in each experimental containing different levels of virgin coconut oil replacing soybean oil.

Fatty acid (g/100 g diet)	SO	LA (15%)	VCO 25%	VCO 50%	VCO 75%	VCO 100%
Caprylic acid (8 : 0)	0.00	0.00	0.21	0.32	0.43	0.54
Capric acid (10 : 0)	0.00	0.00	0.16	0.26	0.37	0.47
Lauric acid (12 : 0)	0.00	1.01	1.53	2.65	4.03	4.92
Myristic acid (14 : 0)	0.00	0.14	0.69	1.04	1.51	1.74
Palmitic acid (16 : 0)	3.93	2.50	3.01	2.55	2.24	1.88
Palmitoleic acid (16 : 1 n7)	0.13	0.09	0.11	0.09	0.08	0.08
Stearic acid (18 : 0)	1.24	0.72	0.78	0.69	0.52	0.46
Oleic acid (18 : 1 n9)	2.60	1.65	2.64	2.21	1.81	1.31
Linoleic acid (18 : 2 n6)	3.74	2.81	3.47	2.58	1.41	0.57
Linolenic acid (18 : 3 n3)	0.30	0.35	0.16	0.14	0.05	0.00

SO: soybean oil; LA: lauric acid; VCO: virgin coconut oil.

**Table 3:** Growth performance of tambaqui juveniles *Colossoma macropomum* submitted to different levels of virgin coconut oil replacing soybean oil during 90 days.

	Soybean oil	LA (15%)	VCO (25%)	VCO (50%)	VCO (75%)	VCO (100%)
BG	587.70 ± 27.10b	573.30 ± 32.50b	671.50 ± 57.40ab	929.80 ± 80.60a	786.30 ± 72.80a	611.90 ± 13.90b
WG	11.2 ± 2.90b	7.30 ± 0.80c	11.90 ± 3.30b	15.40 ± 4.30a	11.80 ± 3.40b	8.30 ± 1.20bc
SG*R*_W_	0.6 ± 0.10a	0.50 ± 0.10a	0.70 ± 0.10a	0.70 ± 0.20a	0.70 ± 0.10a	0.60 ± 0.10a
Kr	1.00 ± 0.01a	0.99 ± 0.01a	1.00 ± 0.01a	1.02 ± 0.01a	1.00 ± 0.01a	1.00 ± 0.02a
*U*_W_	50.00 ± 5.70b	70.00 ± 2.90a	66.10 ± 3.10ab	79.90 ± 7.10a	65.00 ± 5.00ab	55.20 ± 2.70b
AFC	1.40 ± 0.40a	1.90 ± 0.30a	1.20 ± 0.40a	1.10 ± 0.30a	1.40 ± 0.30a	1.80 ± 0.30a

All numeric values in this table were expressed in mean ± standard deviation; LA: lauric acid; VCO: virgin coconut oil; BG: biomass gain (g); WG: weight gain (g); SGR: specific growth rate (%/day); Kr: relative condition factor; *U*_w_: uniformity for weight (%); AFC: apparently feeding conversion; *S*: survival (%). Different letters in the row mean statistical difference by the Tukey test (*p* < 0.05).

**Table 4:** Somatic indices for tambaqui submitted to different inclusions of virgin coconut oil replacing soybean oil and lauric acid after 90 days of feeding.

	Soya oil	LA (15%)	VCO (25%)	VCO (50%)	VCO (75%)	VCO (100%)
VSI	8.03 ± 0.96a	8.19 ± 1.70a	8.03 ± 0.86a	8.59 ± 0.88a	8.58 ± 1.81a	8.65 ± 1.56a
HSI	1.58 ± 0.32a	1.68 ± 2.54a	1.58 ± 0.51a	1.58 ± 0.44a	1.81 ± 0.55a	1.94 ± 0.63a
SSI	0.05 ± 0.01a	0.07 ± 0.03a	0.06 ± 0.03a	0.05 ± 0.01a	0.05 ± 0.01a	0.05 ± 0.01a

All numeric values in this table were expressed in mean ± standard deviation; LA: lauric acid; VCO: virgin coconut oil; different letters in the row mean statistical difference by the Tukey test (*p* < 0.05); VSI: viscerosomatic index; HIS: hepatosomatic index; SSI: spleen somatic index.

**Table 5:** Biochemical blood parameters (mean values ± standard deviation) of tambaqui juveniles *Colossoma macropomum* submitted to different levels of virgin coconut oil replacing soybean oil on feeding trial.

	Soybean oil	LA (15%)	VCO (25%)	VCO (50%)	VCO (75%)	VCO (100%)
Glucose (mg/dL)	41.2 ± 15.5b	43.8 ± 14.2b	59.3 ± 16.9a	52.5 ± 14.2ab	55.3 ± 15.0a	47.7 ± 11.4ab
TPP (mg/dL)	5.58 ± 0.3a	5.43 ± 0.7a	5.36 ± 0.5a	5.53 ± 0.3a	5.56 ± 0.3a	5.61 ± 0.4a
Cholesterol (mg/dL)	188.7 ± 29.6b	170.8 ± 13.7c	186.4 ± 15.5b	181.5 ± 14.6bc	198.8 ± 14.4a	194.3 ± 27.7ab
Triglycerides (mg/dL)	289.6 ± 43.7ab	248.8 ± 72.9bc	256.8 ± 49.2bc	242.4 ± 39.1c	327.0 ± 90.3a	355.4 ± 109.6a

All numeric values in this table were expressed in mean ± standard deviation; LA: lauric acid; VCO: virgin coconut oil; TPP: total plasmatic protein; different letters in the row mean statistical difference by the Tukey test (*p* < 0.05).

**Table 6:** Clinical signs of the tambaqui *Colossoma macropomum* after bacterial challenge with *Aeromonas hydrophila*.

	LA	VCO0%	VCO25%	VCO50%	VCO75%	VCO100%
Lethargy	+	+	-	-	+	+
Hyperemia on skin	+	+	-	-	-	+
Hyperemia on eyes	-	+	-	-	-	+
Melanoma	+	+	-	-	+	+
Rostral hyperemia	-	—	-	-	+	+
Inflamed urogenital opening	-	+	-	-	-	+
Erosion on fins	+	+	-	-	-	+
Ulcerations	+	+	-	-	-	+
Pale gills	-	+	-	-	-	+
Ocular opacity	-	+	-	-	-	+
Hyperemia on fins	+	+	-	-	-	+
Hepatic alterations	+	+	-	-	-	+
Swollen kidney	+	+	-	-	-	+
Swollen spleen	+	+	-	-	-	+
Abdominal distension	-	-	-	-	-	-
Occurrence of signs (%)	64.71	88.24	00.00	00.00	17.64	94.11
Degree of infection	5	5	0	0	3	5

LA: lauric acid; VCO: virgin coconut oil; +: presence of clinical signs; -: absence of clinical signs.

**Table 7:** Biochemical parameters of tambaqui *Colossoma macropomum* after bacterial challenge against *Aeromonas hydrophila*.

	Glu (mg/dL^−1^)	TPP (g/dL^−1^)	Lac (mmol/L)	Hg (g/dL)
FWI	31.67 ± 9.84^b^	4.03 ± 0.70^bc^	2.35 ± 0.68bc	10.41 ± 4.03^a^
FWS	31.00 ± 8.04^b^	3.89 ± 0.64^c^	2.30 ± 0.60bc	10.75 ± 6.74^a^
SO	27.88 ± 14.46^b^	4.17 ± 0.41^bc^	2.50 ± 0.65a	6.50 ± 1.36^ab^
LA	25.67 ± 11.02^b^	3.37 ± 0.47^c^	2.48 ± 0.38a	6.76 ± 1.86^ab^
VCO25%	46.67 ± 15.67^b^	4.33 ± 0.34^ab^	0.91 ± 0.11c	5.53 ± 2.19^b^
VCO50%	41.46 ± 15.48^b^	4.7 ± 0.35^ab^	0.96 ± 0.11c	8.28 ± 2.59^ab^
VCO75%	38.5 ± 18.70^b^	4.38 ± 0.43^ab^	1.78 ± 0.44b	7.73 ± 2.75^ab^
VCO100%	102.50 ± 24.04^a^	5.56 ± 1.38^a^	2.55 ± 0.51a	8.6 ± 2.83^ab^

All numeric values in this table were expressed in mean ± standard deviation; VCO: virgin coconut oil; LA: lauric acid; SO: soybean oil; FWI: fish without injection; FWS: fish with saline solution; Glu: glucose; TPP: total plasmatic protein; Lac: lactate; Hg: hemoglobin; different letters in the column mean statistical difference by the Tukey test (*p* < 0.05).

**Table 8:** Red blood cells, hematocrit, hemoglobin, and hematimetric indices of tambaqui *Colossoma macropomum* after bacterial challenge with *Aeromonas hydrophila*.

	Er (×10^6^)	Ht (%)	MCV (fL)	MCH (pg)	MCHC (%)
FWI	1.75 ± 0.52^a^	18.00 ± 4.00^bc^	137.98 ± 80.59^bc^	69.16 ± 25.04^a^	57.14 ± 21.76^a^
FWS	1.12 ± 0.31^a^	17.00 ± 6.63^bc^	121.21 ± 18.89^bc^	69.49 ± 10.31^a^	57.34 ± 19.80^a^
SO	1.12 ± 0.70^ab^	19.14 ± 3.24^bc^	168.78 ± 45.80^ab^	54.9 ± 12.65^a^	36.53 ± 10.22^abc^
LA	1.02 ± 0.49^b^	13.33 ± 3.05^c^	224.11 ± 75.26^ab^	54.32 ± 20.51^a^	41.10 ± 2.85^ab^
VCO25%	0.80 ± 0.13^b^	17.86 ± 3.34^bc^	173.16 ± 58.42^c^	43.71 ± 28.74^a^	26.49 ± 14.40^bc^
VCO50%	1.39 ± 0.52^a^	23.00 ± 6.58^ab^	216.03 ± 104.66^ab^	56.15 ± 15.58^a^	35.28 ± 15.97^abc^
VCO75%	1.28 ± 0.28^a^	20.00 ± 2.65^c^	185.44 ± 66.48^ab^	53.81 ± 23.37^a^	30.96 ± 11.54^abc^
VCO100%	1.11 ± 0.46^b^	35.67 ± 9.89^a^	279.68 ± 91.21^a^	62.90 ± 20.61^a^	22.04 ± 5.34^c^

All numeric values in this table were expressed in mean ± standard deviation; VCO: virgin coconut oil; LA: lauric acid; SO: soybean oil; FWI: fish without injection; FWS: fish with saline solution; Er: erythrocyte; Ht: hematocrit; MCV: mean corpuscular volume; MCH: mean corpuscular hemoglobin; MCHC: mean corpuscular hemoglobin concentration; different letters in the column mean statistical difference by the Tukey test (*p* < 0.05).

## Data Availability

The data that support the findings of this study are available from the corresponding author upon reasonable request.
